# Optimal Flow Distribution of Military Supply Transportation Based on Network Analysis and Entropy Measurement

**DOI:** 10.3390/e20060446

**Published:** 2018-06-07

**Authors:** Wei Zhou, Jin Chen, Bingqing Ding

**Affiliations:** 1 Business School, Yunnan University of Finance and Economics, Kunming 650221, China; 2 Business School, East China University of Science and Technology, Shanghai 200237, China

**Keywords:** military supply, concealed transportation, flow distribution model, network analysis, entropy measurement

## Abstract

One important element of military supply transportation is concealment, especially during war preparations and warfare periods. By introducing entropy to calculate the transportation concealment degree, we investigate the issue about concealed military supply transportation on the whole road network and propose an optimal flow distribution model. This model’s objective function is to maximize the concealment of military supply transportation. After analyzing the road network, classifying different nodes, summarizing the constraint conditions based on the properties and assumptions in the transportation process, and combining the general parameter limits, the optimal flow distribution model is further transformed into a calculable non-linear programming model. Thus, based on this non-linear programming model, we can obtain the optimal distribution scheme of military supply transportation from the perspectives of network analysis and concealment measurement. Lastly, an example of military supply transportation in Jiangsu province, China is illustrated to prove the feasibility of the proposed model. The managerial implication is that by utilizing the proposed flow distribution model, military supplies can be efficiently transported to the required destinations based on maximizing the concealment degree. Not only this model can be utilized in the real military supply transportation, it can be also applied in other transportation fields which require time efficiency and concealment.

## 1. Introduction

A supply chain enables related enterprises to form a network. This network is significant as it makes enterprises respond promptly according to the dynamic market. Similarly, the supply chains in transport framework also have been broadly discussed. For instance, Mula et al. [1] presented a review of mathematical programming models for supply chain production and transport planning. Xie et al. [2] developed a multistage, mixed inter programming model to fully integrate multimodal transport into the cellulosic biofuel supply chain design under feedstock seasonality. Also, Turki et al. [3] researched the impact of transportation time, remanufacturing cost and configuration of the machines on the optimal capacities of manufacturing stock, purchasing warehouse and the optimal value of returned used end-of-life products. Being different from the traditional transportations, military supply transportation seldom takes cost and time into consideration, but ensures the supply could be delivered to the destination in a safe and concealed way. The key to achieve this goal is to form a concealment military measurement and an optimal flow distribution model that focuses on concealment. However, although there are some methods to measure the concealment, few of them consider networks. As a result, this paper focuses on this vital issue and proposes the concealment measurement of military supply transportation, then constructs the flow distribution model from the perspective of networks.

Some approaches about military supply transportation have been proposed. For example, Cohen [4] provided an empirical approach to analyze the weapon transportation service, Liu and Huang [5] calculated the shortest path of military transportation. However, in our opinion, the shortest path should not be considered as the most important factor in military supply transportation. Similarly, Jain and Saksena [6] explored the optimal route that minimizes military transport time. In order to reasonably transport military supply, Li et al. [7] designed a military supply transportation system, and Montana et al. [8] constructed systematic models to deal with military transport problems. Specifically, Liu et al. [9] gave an information integration model to achieve the uninterrupted connection between military storage and transport, while Montana et al. [8] developed an automatic system for military transportation. Additionally, some recent researches, such as the transportation information support system proposed by Gong et al. [10], attempted to make the military supply delivered automatically. Based on Gong’s system, Dagge et al. [11] constructed military weight transportation systems. Tsadikovich et al. [12] analyzed the military transportation distribution on the basis of the supply-demand relationship. It can be found that the existing models for military supply transportation mostly take road conditions, supply-demand relationship, cost and time into account, but they overlook the transportation concealment.

In addition, some studies attempted to apply the entropy theory to analyze the optimal transportation and flow distribution schedule. For example, Li and Ioannou [13] utilized entropy models to analyze the automation vehicle flow distribution. Also, Yang and Qiu [14] investigated the flow distribution by introducing entropy theory. Actually, the entropy measurement is a famous theory proposed by Abbas [15] and it has been broadly used in many fields, such as energy analysis [16], information clustering [17], accurately prediction [18], image watermarking [19] and mass density measurement [20]. However, it is found that there are only a few researches about military supply based on entropy theory, until Zhou et al. [21] proposed a double-entropy model to address the issue of concealment measurement and detection. This model also reflects that the research neglected the inventory issue in network nodes, while in this study, considering the inventory in network nodes is of a great necessity.

Moreover, many studies about nodes’ inventory of military supply transportation have been investigated by many scholars, such as Bean et al. [22], Taleizadeh et al. [23] and Yang et al. [24]. Some useful methods are as follows: Liu and Lian [25] studied the optimal inventory with respect to the minimized cost, Fiestras-Janeiroabcd [26] used the cooperative game theory to manage the military supply inventory, Ventura et al. [27] explored a nonlinear programming model to determine the optimal inventory, and Parhizkari et al. [28] used Non-dominated Sorting Genetic Algorithm-II (NSGA-II) as meta-heuristic technique and examined the validity of the proposed inventory management model. Recently, Russell and Urban [29] developed a mixed-integer programming model through applying symmetry-breaking constraints to calculate the optimal inventory, and Firouz et al. [30] proposed the multi-sourcing and lateral transshipment method to analyze the integrated inventory. It should be pointed out that these inventory controlling models are not suitable for military supply transportation and inventory analysis. Actually, the military supply inventory is different from other existing studies as it is not for economic benefits, but for minimizing the time gap between the steps of material preparation and material supply. In this paper, different types of node inventories are analyzed in detail because a huge amount of military supplies are needed when there is a war, setting inventories in network nodes is the most direct and efficient way to ensure military supplies could be provided in time. Despite a wide range of publications related to the inventory of military supply, little is actually known about considering time efficiency and concealment. As a result, this study is attempting to bridge the research gap between entropy theory and transportation concealment measurement based on the nodes’ inventory in road networks.

The main contribution of this paper is in adapting the optimal flow distribution model to fit the context of military supply transportation based on entropy theory. By maximizing the concealment degree, military supplies can be transported to the required destinations safely and efficiently. As aforementioned, other relevant methods mainly consider the time and cost the transport will take, but they are not in the scope of consideration in this study. Instead, concealment is believed to be the most significant objective due to the special characteristics of military supply transportation. Thus, in this paper, we investigate the military supply concealment on the aspect of transport volume in the nodes of military networks. After that, we construct a military supply transportation concealment measurement and an optimal flow distribution model by introducing entropy theory. Lastly, an example is provided to illustrate the validity and feasibility of the proposed model.

To do so, this paper proposes the theoretical evidences and the concealment measurement of military supply transportation in Section 2. The concealment measurement equations and the optimal flow distribution model for different roads are constructed in Section 3. Besides, the proposed model’s related properties are analyzed. In Section 4, an example is illustrated to prove the model’s feasibility and effectiveness. Finally, this paper ends up with some conclusions in Section 5.

## 2. Entropy Measure for the Concealment of Military Supply Transportation

Due to the fact that military supplies are significant and confidential, the transportation is required to be concealed. Some studies attempted to apply entropy to measure the concealment of military supply transportation which was proved to be feasible [21], because entropy can measure the uncertainties such as “dispersion degree” and “disorder degree” in the transportation. According to the prior studies about entropy measure approaches for military supply concealed transportation, this paper considers different nodes in military transport networks, constructs a new entropy measure and develops an optimal flow distribution model which is analyzed based on some general conditions in military supply transportation. To make it clear, all decision variables are summarized in Table 1.

Here are some basic concepts for the model:

If $R\left(i,j\right)$ represents the number of road sections from *i* to *j*, $r\left(i,j\right)$ denotes one of the road sections, then the traffic flow on each road section and the total traffic flows can be defined as $F_{i,j}^{r(i,j)}$ and *F*. Moreover, let $s_{i,j}^{r(i,j)}$ be the length of the road $r\left(i,j\right)$ from *i* to *j* and $y_{i,j}^{r(i,j)}$ be the standard traffic flows which are the ones of the normalized road sections and its interval value is [0,1]. Thus, we can find that there are $R\left(i,j\right)$ roads from *i* to *j* and the total number of military supplies from the starting point is 1. Additionally, $y_{i,j}^{r(i,j)}$ represents the final distributed flows of road $r\left(i,j\right)$ from *i* to *j* and it ris an unknown parameter calculated by the optimal flow distribution model proposed in next section.

According to the definition of information entropy made by Shannon [31] and Abbas [15], Yang and Qiu [14] theoretically analyzed the information entropy with traffic flow, and Zhou et al. [21] applied the information entropy to investigate the concealment of military supply transportation. Based on the prior research findings, the general concealment equation can be constructed as I(x)=−kxln(x) which is utilized to measure the concealment of the standard traffic flow *x* on a single-lane road in the unit distance. In this equation, the *k* is the *Boltzmann Constant* and k≥0. To deal with the problem that the network concealment cannot be efficiently identified with this equation when the road conditions are different for their width and length, the number of lanes are increased to *q* and the length of the road is *s*. Therefore, the network concealment measurement equation of the standard flow *x* in all road conditions can be further defined as I(x)=ksx(−lnx+lnq).

Compared with the original one, the above equation takes all the road conditions into consideration so that it can efficiently identify the concealment in different road conditions, but this concealment measure equation is non-monotonic. Meanwhile, the final distribution plan determined by the directionality of supply transport in the original network is non-optimal. To address these issues and take flow limitations into account, we develop the following modified concealment measure model which is more systematic and accord with the reality. More importantly, it is able to provide a reasonable flow distribution scheme when maximizing the concealment. This modified concealment measure model is shown below.

(1) $$I\left(x\right)=ks_{i,j}^{r(i,j)}\times \left(y_{i,j}^{r(i,j)}\left(\ln q_{i,j}^{r(i,j)}-\ln y_{i,j}^{r(i,j)}\right)+y_{j,i}^{r(j,i)}\left(\ln q_{j,i}^{r(j,i)}-\ln y_{j,i}^{r(j,i)}\right)\right)$$

Equation (1) is our objective function to maximize the concealment of one road from *i* to *j*. Meanwhile, the equation considers the non-directionality of the road. There are some conclusions on the basis of the above concealment measure method: (1) a longer road section determines a greater concealment degree; (2) an increasing number of lanes determine a greater concealment degree. According to these conclusions, Equation (1) can reasonably calculate the concealment of military supply transportation. Meanwhile, the constraints of different nodes in military transport networks are considered to make the proposed model accord with the reality. Some detailed analyses about the network nodes in military supply transportation, road section concealment and road network properties will be discussed in the next section. Further, the new optimal flow distribution model in military networks will be constructed.

## 3. Network Analyses of Military Supply Concealment Transportation

To efficiently transport military supplies, some constraints of different nodes are involved. The traffic flows in the whole military road networks could be adjusted by changing the quantity of supplies in the nodes. Thus, this section will analyze the specific types of nodes in the transportation, related concealment measure, and military road network properties.

### 3.1. The Road Network Nodes and Concealment Analyses of Military Supply Transportation

As mentioned before, setting inventories in network nodes is the most direct and effective way to ensure military supplies could be provided in time. As a result, the inventories in road network nodes are significant for the national defense and army’s combat power. Hence, this paper analyzes the node types and concealment in related road sections in the following content.

**Figure 1:**
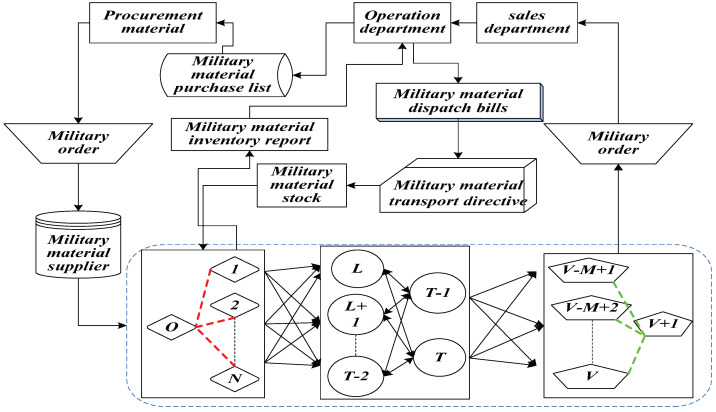
The road network of military supply transportation.

As shown in Figure 1, military supplies are delivered stage by stage from the starting point of sending orders to the endpoint. This paper mainly analyzes the content highlighted with a dotted line which represents the process that military supplies are transported from the starting point to the endpoint. This process is explained as follows: military supplies are firstly delivered from the points between 1 and *N* to the points between *L* and *T*. Also, the military supplies can be transported in the points between *L* and *T* mutually without limit of directions. Then, the military supplies are transported to the points between V−M+1 and *V*. Through the analysis, there are three different types of road network nodes which are the starting points from 1 to *N* , the transit points from *L* to *T* and the endpoints from V−M+1 to *V*. Different nodes have their own inventories and different inventories have their own constraints.

This study uses qualitative and quantitative ways to analyze different types of military network nodes. First, the nodes’ properties and the relationship between this network and its surrounding networks are analyzed qualitatively to clarify different functions of the nodes. After that, each node and its related road section’s concealment measure equation is proposed by a quantitative analysis according to the changes of node inventories and transport volume and directions. To effectively identify the rest of inventories in different points, $g_{j}$ is introduced to represent the initial inventory in point *j*, $t_{1}^{j}$ denotes the amount of supplies delivered to the point *j* and $t_{2}^{j}$ denotes the amount of supplies delivered out from the point *j*.

**Definition** **1.** *In the military supply road network, if there are supplies transported out from the point j but no supplies transported to this point, then we have $y_{\left(j,t\right)}^{r(j,t)}\geq 0$, $y_{\left(t,j\right)}^{r(t,j)}=0$, and t∈(1,2⋯,V+1), j=0. For the points like this, we identify them as starting points.*

**Figure 2:**
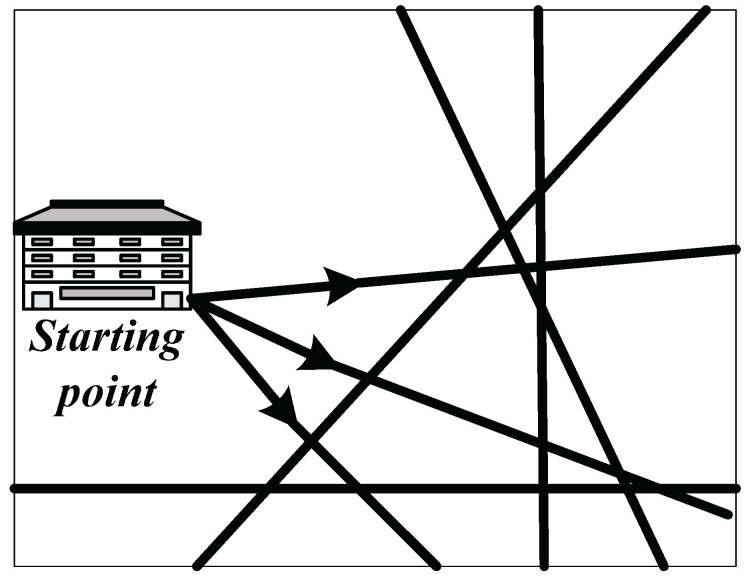
The starting point in military supply transportation.

It can be seen from Figure 2 that military supplies are transported from the starting point. The distance between the starting point and endpoint is long. Therefore, the final inventory in the starting point can be set as zero which means all the supplies are transported to the transit points and endpoint. It helps if the supplies could be delivered to the battlefield through the transit points and endpoint as soon as possible and ensures enough supplies needed by various military departments could be transported in time. Thus, the constraint equation of the starting point is:

(2) $$g_{0}-\sum_{j=1}^{V+1}y_{0,j}^{r(0,j)}=0$$

In Equation (2), $g_{0}$ represents the initial inventory in the starting point. It can be seen from Figure 1 that there are N multi-starting points and M multi-endpoints which are mentioned before. By increasing the virtual starting points 0 and endpoints V+1, the multi-starting points and multi-endpoints can be transferred into single starting points and endpoints. The distance of the roads that connect the virtual points and actual starting points, and the roads that connect each virtual point is 0. Therefore, the above situation does not affect the degree of concealment of the whole military supply transportation.

The supply transport from the starting point to transit points and endpoint is directional because if it is a bi-directional transport, there will be circular flows in the road network which leads the increase of traffic flows and decrease of concealment degree. Therefore, the concealment measure equation of the road sections connected with the starting point can be presented as:

(3) $$I\left(y\right)=k\sum_{j=1}^{V+1}\sum_{r(0,j)}^{R(0,j)}s_{0,j}^{r(0,j)}y_{0,j}^{r(0,j)}\left(\ln q_{0,j}^{r(0,j)}-\ln y_{0,j}^{r(0,j)}\right)$$

**Definition** **2.** *In the military supply road network, if there are military supplies transported in and out from the point j, then we have $y_{\left(i,j\right)}^{r(i,j)}\geq 0$, $y_{\left(j,t\right)}^{r(j,t)}\geq 0$, and i∈(0,1,⋯,V), j∈(1,2,⋯,V), t∈(1,2,⋯,V+1). For the points like this, we define them as transit points.*

**Figure 3:**
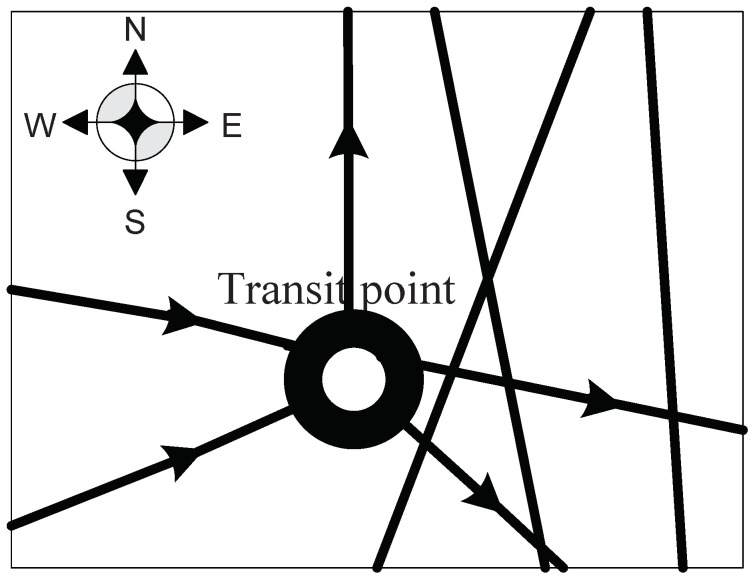
The transit point that inflows are less than the outflows.

**Figure 4:**
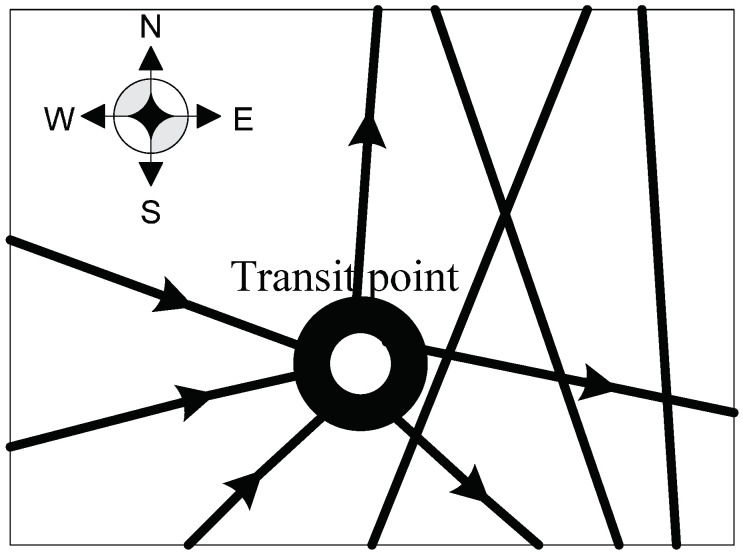
The transit point that inflows are equal to the outflows.

**Figure 5:**
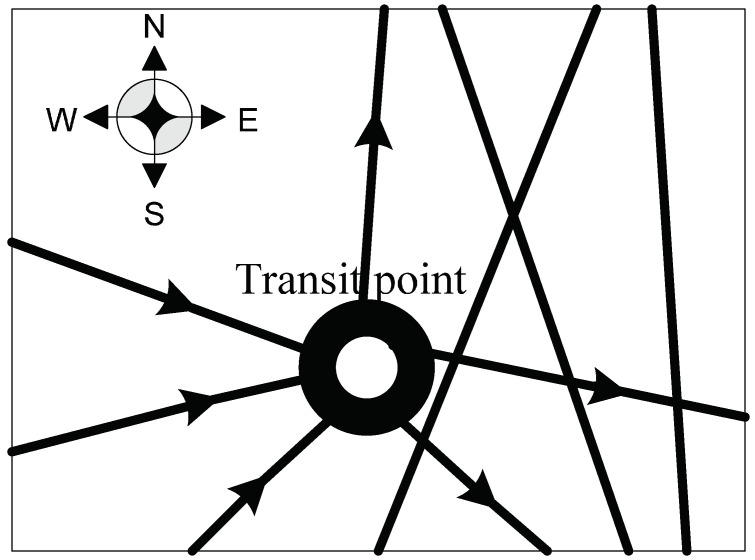
The transit point that inflows are more than the outflows.

Transit points could be anywhere along the route. Therefore, setting inventories in transit points is important to ensure military supplies could be provided in time. It is found that every node’s maximum capacity is not bigger than 1 and the actual inventory depends on the real needs of military supplies during the warfare. The transit point has its own initial inventory. When the military supplies are transported from the starting point to the transit point, there should be three situations:

(1) $g_{j}+t_{1}^{j}-t_{2}^{j}\leq g_{j}$, the equation shows that the final inventory in transit point is less than the initial one. The number of military supplies transported to the transit point is smaller than the one transported out which is illustrated by the number arrows in Figure 3. Here, we can find that the inflows are less than the outflows. This situation happens because the military supplies that transported out from the transit point consist of the supplies delivered from the starting point and the ones that are already in the initial inventory. Hence, the number of the military supplies transported in the transit point is smaller than the one transported out. The transit point might be far from the endpoint, so the military supplies are delivered from this transit point to another one near it or to the endpoint directly for convenience. Meanwhile, the road section connecting the transit point and another one or the endpoint has a large transport capacity, which allows the military supplies transported from the transit point to another one or the endpoint. Therefore, we have:

(4) $$\sum_{i=0}^{V}y_{i,j}^{r(i,j)}+g_{j}-\sum_{i=0}^{V}y_{j,i}^{r(j,i)}<g_{j},\;j\in \left(1,\cdots ,V\right)$$

(2) $g_{j}+t_{1}^{j}-t_{2}^{j}=g_{j}$, the equation shows that the final inventory in transit point is equal to the initial one. As mentioned before, the number of arrows in Figure 4 refers to the amount of military supplies transported in and out from the transit point. Similarly, it can be seen from Figure 4 that the inflows are equal to the outflows. The military supplies are transported in the transit point, and then they are delivered to another transit point or the endpoint. This transit point is far from the endpoint which possibly makes it difficult for the military supplies to be transported to the destination in time, especially when the traffic situation is influenced during the warfare. Once the road is clear, the military supplies will be sent as soon as possible. Therefore, the inventories could be set in this kind of transit points. So we have:

(5) $$\sum_{i=0}^{V}y_{i,j}^{r(i,j)}+g_{j}-\sum_{i=0}^{V}y_{j,i}^{r(j,i)}=g_{j},\;j\in \left(1,\cdots ,V\right)$$

(3) $g_{j}+t_{1}^{j}-t_{2}^{j}>g_{j}$, the equation shows that the final inventory in transit point is more than the initial one. According to Figure 5, the number arrows illustrates that the inflows are more than the outflows, which is to say the military supplies transported to the transit point are more than which transported out. This situation happens because a part of the military supplies transported to the transit point are delivered out and the rest are left in the transit point which increases the inventory in the transit point. This transit point is near the endpoint and the road section is of a great concealment. Thus, even though an emergency happens, the military supplies could be sent to the destination in time. However, the traffic flow on this road section might be small, so there should be more inventories in this kind of transit points to make the military supplies well prepared. Then, we have:

(6) $$\sum_{i=0}^{V}y_{i,j}^{r(i,j)}+g_{j}-\sum_{i=0}^{V}y_{j,i}^{r(j,i)}>g_{j},\;j\in \left(1,\cdots ,V\right)$$

Due to the reason that military supply transportation is urgent and crucial, the road sections connecting each transit point are non-directional. To transport the military supplies to the destination with the least possible delay, obeying the traffic rules is relatively unnecessary. However, the final transportation is directional. Because the road sections which connect transit nodes are non-directional, the second part of the network’s concealment measure equation is:

(7) $$I\left(y\right)=k\sum_{j=1}^{V}\sum_{i=1}^{V}\sum_{r(i,j)}^{R(i,j)}\left(s_{i,j}^{r(i,j)}y_{i,j}^{r(i,j)}\left(\ln q_{i,j}^{r(i,j)}-\ln y_{i,j}^{r(i,j)}\right)+s_{j,i}^{r(j,i)}y_{j,i}^{r(j,i)}\left(\ln q_{j,i}^{r(j,i)}-\ln y_{j,i}^{r(j,i)}\right)\right)$$

**Definition** **3.** *I In the military supply road network, if there are military supplies transported to the point j but no military supplies transported out from it, then we have $y_{\left(i,j\right)}^{r(i,j)}\geq 0$, $y_{\left(j,i\right)}^{r(j,i)}=0$, and i∈(0,1,2,⋯,V), j=V+1. For the points like this, we define them as endpoints.*

**Figure 6:**
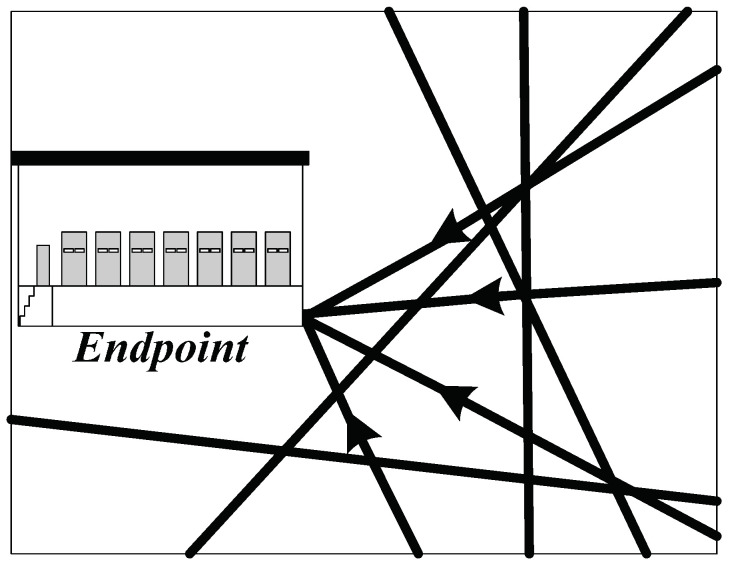
The end point in military supply transportation.

In the military supply road network, an endpoint is also the destination where military supplies should be transported to. As shown in Figure 6, this point only receives military supplies and there is no outflow. Once there is a war, before making the road clear, it is vital to ensure plenty of military supplies are prepared in the endpoint, so that it will not significantly influence the operation of national economy. At the same time, the combat equipment should be provided to the army without interruption. Thus, there should be a huge inventory setting in the endpoint. The amount of this inventory is between the minimum limit and the maximum limit of 1. Thus, we have the constraint equation of the endpoint as follows:

(8) $$1\geq \sum_{i=0}^{V}y_{i,V+1}^{r(i,V+1)}+g_{V+1}\geq Min$$

There are some similarities between the concealment measure equations of the starting point and the endpoint. Both of their transportations are directional. As a result, the concealment measure equation of the road sections that connect the endpoint is:

(9) $$I\left(y\right)=k\sum_{i=0}^{V}\sum_{r(i,V+1)}^{R(i,V+1)}s_{i,V+1}^{r(i,V+1)}y_{i,V+1}^{r(i,V+1)}\left(\ln q_{i,V+1}^{r(i,V+1)}-\ln y_{i,V+1}^{r(i,V+1)}\right)$$

From the view point of networks, besides the capacity constrains in the whole network nodes, the supply amount of the entire road network should satisfy the conservation law, which is formed as the equation below:

(10) $$1+\sum_{j=0}^{V+1}g_{j}=\sum_{j=0}^{V+1}(\sum_{i=0}^{V}y_{i,j}^{r(i,j)}+g_{j}-\sum_{i=1}^{V+1}y_{j,i}^{r(j,i)})$$

By setting inventories in network nodes, the military supplies could be prepared in advance, which saves a lot of manpower, energy and economic resources. Thus, no matter when a war breaks out, there is more manpower can be assigned to complete other tasks and the pressure of production will be reduced. As a result, this paper proposes a new and modified concealment measure equation which considers node capacity constraints. Further, in order to construct a optimal flow distribution model that maximizes the concealment degree, the following part will analyze the network properties of military supply transportation.

### 3.2. The Network Properties of Military Supply Transportation

Based on the existing concealment measure model, the new constructed model owns some general properties which also belong to the existing ones. The properties are introduced briefly as follows:

**Property** **1.** *The military supply transportation in the road sections that connect the starting point and endpoint are directional.*

Property 1 means the starting point should only transport the military supplies out so there are only outflows from this point, while the endpoint should only receive the military supplies so there are only inflows to this point. If the directions are reversed, there will be circular flows in the road network, leading the increase of traffic flow and decrease of concealment degree.

**Property** **2.** *In the real situation, there is only one direction in the transportation of transit roads that are located between the starting point and the end point.*

Property 2 could be described as the equation $y_{i,j}^{r(i,j)}\times y_{j,i}^{r(i,j)}=0$. If it is bi-directional, there will be circular flows in the road network which leads the decrease of concealment degree.

**Property** **3.** *The circular flows will exist when calculating the optimal flow distribution which maximizes the concealment degree.*

Due to the monotonicity of the concealment measure and diversity of the road network, there might be some inefficient distribution schemes like circular flows in the optimal flow distribution results. Thus, Property 3 shows a possible phenomenon in the calculation process. A handing approach is proposed in the next section.

Besides the general properties, some new properties caused by different types of nodes are summarized and analyzed as follows:

**Property** **4.** *The final amount of military supplies in the starting point is zero.*

This property shows that all the military supplies in the starting point are translated out. The amount of these military supplies also meets the optimal goal. Of course, in the real application, the starting point can hold some military supply which is uninfluential for the optimal schedule based on the proposed model in this paper.

**Property** **5.** *After transporting in and out the military supplies, the amount of military supplies in each transit point is non-negative and should not exceed the maximum capacity.*

According to Property 6, we can get $g_{i}\geq 0$ and $1\geq g_{i}+t_{1}^{i}-t_{2}^{i}\geq 0$.

**Property** **6.** *The amount of the military supplies in the starting point and transit points should equal to the amount of military supplies in all of the nodes after the transport process.*

**Proof** **of** **Property** **6.** $g_{j}^{\prime }$ is introduced to represent the final inventory in node *j*, and $g_{j}$ refers to the initial inventory in node *j*. $t_{1}^{j}$ refers to the amount of the military supplies transported to node *j*, and $t_{2}^{j}$ refers to the amount of the military supplies transported out from node *j*. Through analyzing a node’s transport volume, we have the equation $g_{j}+t_{1}^{j}=g_{j}^{\prime }+t_{2}^{j}$. It could be found in this node that the initial inventory plus the military supplies transported to the node equals to the final inventory plus the military supplies transported out from the node. By considering the changes happen in all the road network nodes, we have:

(11) $$\sum_{j=0}^{V+1}\left(g_{j}+t_{1}^{j}\right)=\sum_{j=0}^{V+1}\left({g^{\prime }}_{j}+t_{2}^{j}\right)$$

In the equation, $\sum_{j=0}^{V+1}\left(g_{j}+t_{1}^{j}\right)=\sum_{j=0}^{V+1}g_{j}+\sum_{j=0}^{V+1}t_{1}^{j}$. All the military supplies are transported from the starting point and the amount is 1. Therefore, we have $\sum_{j=0}^{V+1}\left(g_{j}+t_{1}^{j}\right)=\sum_{j=0}^{V+1}g_{j}+1$. Similarly, all the military supplies will be transported to the transit points and endpoint to become their inventories ultimately, which means there is no military supply on the road sections. As a result, we have the total transport volume equation $\sum_{j=0}^{V+1}g_{j}+1$, $\sum_{j=0}^{V+1}{g^{\prime }}_{j}$ which satisfies the equilibrium of the total transport volume. ☐

These results will be utilized in the following constraint constructions and model calculation. By analyzing the road network, its nodes and the corresponding properties in the process of military supply transportation, some constraints are obtained for the new concealment measure, which enables the traffic flows to be distributed reasonably. Additionally, this new model also has the advantage to measure the concealment degree which makes it more practical than other military supply transportation models.

## 4. Optimal Flow Distribution Model of Military Supply Transportation in the Road Network

The constraint conditions could be obtained by analyzing the road network, its properties, and the changes in the transportation volume, which will be utilized in the general concealment measure model. In the proposed model, different types of nodes and transportation relationships in the road sections are involved. Meanwhile, the optimal flow distribution model of military supply transportation is constructed.

### 4.1. Assumptions for the Optimal Flow Distribution Model

Through the above analyses, the concealment measure based on entropy theory satisfies the requirements between the relationship of the transportation concealment and the transportation volume. To further construct the optimal flow distribution model of military supply transportation, some assumptions are set as follows:

**Assumptions** **1.** *In the military supply transportation, the capacity and distance of the road sections can be known and the transportation expenses are not in the scope of consideration.*

**Assumptions** **2.** *The main goal of the military supply transportation is to maximize the concealment degree.*

**Assumptions** **3.** *The transportation capacities in all the road sections are the same.*

**Assumptions** **4.** *When the military supplies are transported on multi-lanes, they are divided equally on each of the lane.*

According to the above assumptions, there is a positive relationship among the concealment degree, the number of lanes on the road sections and the length of the road. Combining with the assumptions and the properties of the road network, the specific constraints could be obtained which could be seen from the illustrative example.

Based on the Property 2, there is only one direction in the road sections between the starting point and the endpoint. Meanwhile, there only should be outflows from the starting point and inflows to the endpoint. The amount of the military supplies transported from the starting point is 0. On the basis of these conditions, we can get the following constraint:

(12) $$\sum_{j=1}^{V+1}y_{0,j}^{r(0,j)}=1,y_{ij}^{r(i,j)}\times y_{ji}^{r(j,i)}=0$$

Then, R(i,j) is the amount of road sections from point *i* to point *j*. There are more than one road sections and the road section is non-directional. According to the requirement of maximizing the objective function under the constraint conditions, the new concealment measure equation and optimal flow distribution model are constructed. Further, through considering the circulation flows, we have Equations (13) and (14).

(13) $$r\left(i,j\right)=r\left(j,i\right),s_{i,j}^{r(i,j)}=s_{j,i}^{r(j,i)},q_{i,j}^{r(i,j)}=q_{j,i}^{r(j,i)}$$

(14) $$y_{i,j}^{r(i,j)}\times y_{j,t}^{r(j,t)}\times \cdots \times y_{l,i}^{r(l,i)}=0$$

The proposed method in this paper removes the circulations and saves the fitting times from the beginning of constructing the model. Therefore, the general constraint conditions of concealment measure could be obtained. The next section introduces the optimal flow distribution model according to the above objective function and constraint conditions.

### 4.2. The Optimal Flow Distribution Model of Military Supply Transportation

As mentioned before, the network of military supply transportation could be divided into three parts. The first and the third parts are directional (see Property 1). The second part consists of the road sections between the starting point and the endpoint with uncertain transport directions. The objective function of military supply transportation is formed and applied to the transportation which involves the inventory. By analyzing the concealment measures of different road sections namely Equations (3), (7) and (9), we can obtain Equation (13) which is a general concealment measure of military supply transportation based on the road networked.

(15) $$I\left(X\right)=maxk\left\{\begin{array}{c} \sum_{j=1}^{V+1}\sum_{r(0,j)}^{R(0,j)}s_{0,j}^{r(0,j)}y_{0,j}^{r(0,j)}\left(\ln q_{0,j}^{r(0,j)}-\ln y_{0,j}^{r(0,j)}\right)+\\ \sum_{j=1}^{V}\sum_{i=1}^{V}\sum_{r(i,j)}^{R(i,j)}\left(\begin{array}{c} s_{i,j}^{r(i,j)}y_{i,j}^{r(i,j)}\left(\ln q_{i,j}^{r(i,j)}-\ln y_{i,j}^{r(i,j)}\right)+\\ s_{j,i}^{r(j,i)}y_{j,i}^{r(j,i)}\left(\ln q_{j,i}^{r(j,i)}-\ln y_{j,i}^{r(j,i)}\right) \end{array}\right)\\ +\sum_{i=0}^{V}\sum_{r(i,V+1)}^{R(i,V+1)}s_{i,V+1}^{r(i,V+1)}y_{i,V+1}^{r(i,V+1)}\left(\ln q_{i,V+1}^{r(i,V+1)}-\ln y_{i,V+1}^{r(i,V+1)}\right) \end{array}\right\}$$

By considering the general properties mentioned in Section 3.1 such as Properties 1, 2, 3 and combining with Equations (13) and (14), we have the general constraint conditions of the military supply transportation, which can be presented as Equations (16)–(18).

(16) $$y_{i,0}^{r(i,0)}=0,y_{V+1,i}^{r(v+1,i)}=0,\sum_{j=1}^{V+1}y_{0,j}^{r(0,j)}=1;$$

(17) $$y_{ij}^{r(i,j)}\times y_{ji}^{r(j,i)}=0,i,j\in \left(0,1,2,\cdots ,V+1\right);$$

(18) $$r\left(i,j\right)=r\left(j,i\right),s_{i,j}^{r(i,j)}=s_{j,i}^{r(j,i)},q_{i,j}^{r(i,j)}=q_{j,i}^{r(j,i)};$$

After that, the new constraints Equations (19)–(24) in the concealment measure could be obtained according to the new properties caused by different types of nodes such as Property 4, 5 and 6.

(19) $$1+\sum_{j=0}^{V+1}g_{j}=\sum_{j=0}^{V+1}(\sum_{i=0}^{V}y_{i,j}^{r(i,j)}+g_{j}-\sum_{i=1}^{V+1}y_{j,i}^{r(j,i)})$$

(20) $$g_{0}-\sum_{j=1}^{V+1}y_{0,j}^{r(0,j)}=0$$

(21) $$1\geq \sum_{i=0}^{V}y_{i,V+1}^{r(i,V+1)}+g_{V+1}\geq Min$$

(22) $$\sum_{i=0}^{V}y_{i,j}^{r(i,j)}+g_{j}-\sum_{i=0}^{V}y_{j,i}^{r(j,i)}>g_{j},\;j\in \left(1,2,\cdots ,V\right)$$

(23) $$\sum_{i=0}^{V}y_{i,j}^{r(i,j)}+g_{j}-\sum_{i=0}^{V}y_{j,i}^{r(j,i)}=g_{j},j\in \left(1,2,\cdots ,V\right)$$

(24) $$\sum_{i=0}^{V}y_{i,j}^{r(i,j)}+g_{j}-\sum_{i=0}^{V}y_{j,i}^{r(j,i)}<g_{j},j\in \left(1,2,\cdots ,V\right)$$

In conclusion, the optimal flow distribution model for different types of nodes is constructed based on the objective function of the military supply transportation, namely Equation (15), and the network constraint conditions, namely Equations (16)–(24). The application of this model and the calculation process are illustrated in the next section.

## 5. Illustrative Example

After introducing the concealment measure and the related theories and properties of optimal flow distribution model, this part illustrates the feasibility and effectiveness of the proposed model. Even though there is no strict war around us, it is necessary to have military exercises. As a result, the concealed and efficient military supply transportation is significant. Take the defense works in Shandong, China as an example, as a province with abundant resources, Jiangsu needs to transport a huge amount of military supplies to Shandong. Nanjing is selected to be the starting point which is the capital of Jiangsu province, while other cities in Jiangsu are the transit points and two of them are the endpoints. Meanwhile, some high ways are chosen to be the transport road sections, hence the concealment measure and optimal flow distribution model proposed in this paper is select to model and obtain the optimal transportation schedule.

### 5.1. Background and Calculation

Suppose that the military supplies are transported from Nanjing, Jiangsu to the boundary between Jiangsu and Shandong. When the military supplies are sent from Nanjing, they are transported though Wuxi, Yangzhou, Zhenjiang and Changzhou to Suqian, Huaian and Yancheng.

**Figure 7:**
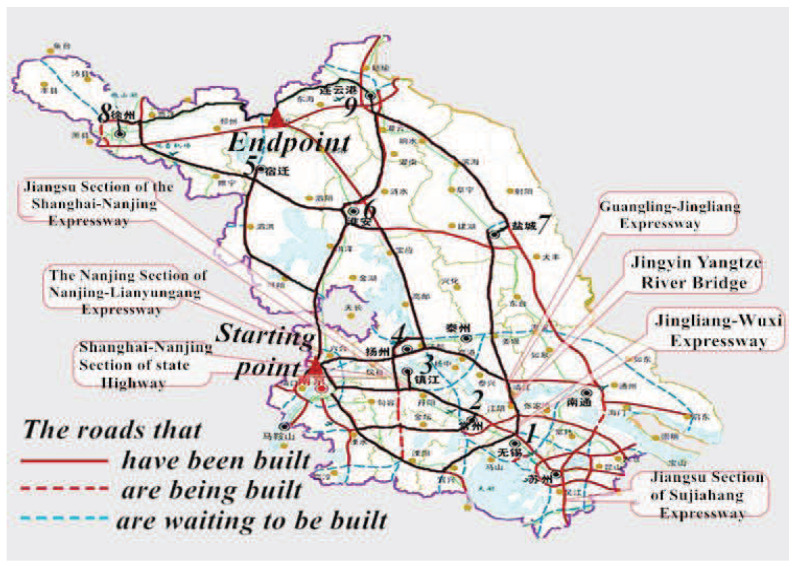
The high ways in Jiangsu province.

Finally, they arrive in Lianyungang and Xuzhou, which are the military supply storage sites and also the endpoints. Road transportation is the main transport approach as air and railway transportations are easy to be noticed by the enemy. Therefore, the high ways that have already been built are selected to be the road sections of the transportation. The specific route could be seen from Figure 7 which is highlighted with black lines.

**Figure 8:**
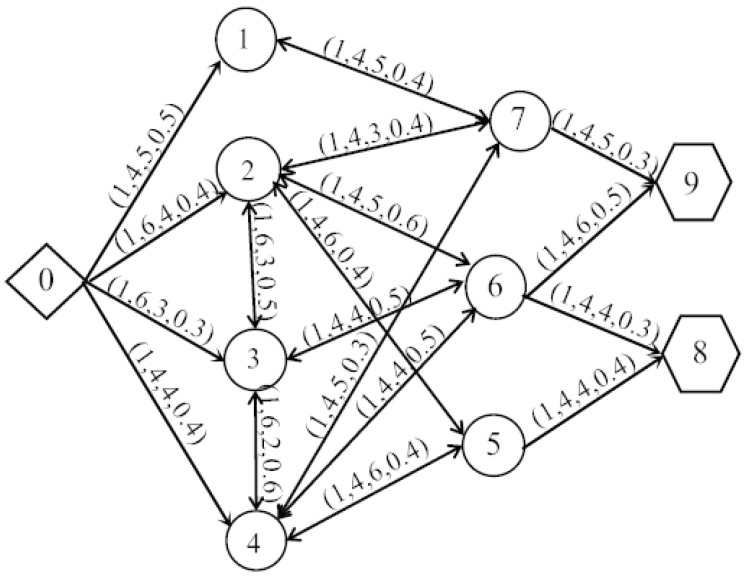
Military transportation road network.

Suppose that there is a huge amount of military supplies waiting to be transported from Nanjing Military Region to the military supply storage sites which are in the boundary between Jiangsu and Shandong, some typical roads are chosen to be the example to illustrate the transportation. The road network could be seen from Figure 8. There are different shapes represent different types of the nodes. For instance, the rhombus is the starting point and the circles are the transit points, while the pentagons are the endpoints. Also, the related elements about the road network are labeled with some symbols (r,q,s,h). Here, *r* means the road sections which connect two places, *q* is the number of lanes, *s* is the length of a road section and *h* is the maximum traffic flow. For example, a road section from 0 to 1 could be represented as (1, 4, 5, 0.5), which means it is the first road section from 0 to 1, there are 4 lanes, the length of the road section is 5 and the maximum military supply traffic flow is 0.5.

Besides, the amount of military supplies that already in each point is *G*. Here, we set *G* as $\left(g_{1},g_{2},g_{3},g_{4},g_{5},g_{6},g_{7},g_{8},g_{9}\right)=\left(0.15,0.20,0.20,0.20,0.1,0.05,0.05,0,0\right)$. Suppose that the inflows are equal to the outflows in transit point 1 and the maximum inventories Max in transit points 2, 3 and 4 are: (0.15, 0.15, 0.15). The minimum inventories Min in the transit points 5, 6 and 7 and the endpoints 8 and 9 should not be less than (0.20, 0.15, 0.15, 0.60, 0.50). Due to the change of inflows and outflows in the nodes, the rest of military supplies in each node are changed.

Based on the objective function of military supply transportation, namely Equation (15), the objective function of this example can be formed as below:

(25) $$\max\left\{\begin{array}{c} 5\left[y_{0,1}^{1}\left(ln4-lny_{0,1}^{1}\right)\right]+4\left[y_{0,2}^{1}\left(ln6-lny_{0,2}^{1}\right)\right]+3\left[y_{0,3}^{1}ln6-lny_{0,3}^{1})\right]+4\left[y_{0,4}^{1}\left(ln4-lny_{0,4}^{1}\right)\right]+\\ \left(\begin{array}{c} 3\left[y_{2,3}^{1}\left(ln6-lny_{2,3}^{1}\right)\right]+3\left[y_{3,2}^{1}\left(ln6-lny_{3,2}^{1}\right)\right]+2\left[y_{3,4}^{1}\left(ln6-lny_{3,4}^{1}\right)\right]+2\left[y_{4,3}^{1}\left(ln6-lny_{4,3}^{1}\right)\right]\\ +5\left[y_{1,7}^{1}(\left(ln4-lny_{1,7}^{1}\right)\right]+5\left[y_{7,1}^{1}\left(ln4-lny_{7,1}^{1}\right)\right]+3\left[y_{2,7}^{1}\left(ln4-lny_{2,7}^{1}\right)\right]+3\left[y_{7,2}^{1}\left(ln4-lny_{7,2}^{1}\right)\right]\\ +5\left[y_{2,6}^{1}\left(ln4-lny_{2,6}^{1}\right)\right]+5\left[y_{6,2}^{1}\left(ln4-lny_{6,2}^{1}\right)\right]+6\left[y_{2,5}^{1}\left(ln4-lny_{2,6}^{1}\right)\right]+6\left[y_{5,2}^{1}\left(ln4-lny_{5,2}^{1}\right)\right]\\ +4\left[y_{3,6}^{1}\left(ln4-lny_{3,6}^{1}\right)\right]+4\left[y_{6,3}^{1}\left(ln4-lny_{6,3}^{1}\right)\right]+5\left[y_{4,7}^{1}\left(ln4-lny_{4,7}^{1}\right)\right]+5\left[y_{7,4}^{1}\left(ln4-lny_{7,4}^{1}\right)\right]\\ +4\left[y_{4,6}^{1}\left(ln4-lny_{4,6}^{1}\right)\right]+4\left[y_{6,4}^{1}\left(ln4-lny_{6,4}^{1}\right)\right]+6\left[y_{4,5}^{1}\left(ln4-lny_{4,5}^{1}\right)\right]+6\left[y_{5,4}^{1}\left(ln4-lny_{5,4}^{1}\right)\right] \end{array}\right)\\ +5\left[y_{7,9}^{1}\left(ln4-lny_{7,9}^{1}\right)\right]+6\left[y_{6,9}^{1}\left(ln4-lny_{6,9}^{1}\right)\right]+4\left[y_{6,8}^{1}\left(ln4-lny_{6,8}^{1}\right)\right]+4\left[y_{5,8}^{1}\left(ln4-lny_{5,8}^{1}\right)\right] \end{array}\right\}$$

According to the aforementioned constraint conditions, namely Equations (16)–(24), the constraint conditions of the optimal flow distribution model in this example can be presented as follows:

(26) $$s.t.\left\{\begin{array}{c} y_{0,1}^{1}+y_{0,2}^{1}+y_{0,3}^{1}+y_{0,4}^{1}=1;y_{7,9}^{1}+y_{6,9}^{1}\geq 0.50;y_{6,8}^{1}+y_{5,8}^{1}\geq 0.6;\\ y_{0,1}^{1}+y_{7,1}^{1}+0.15-y_{1,7}^{1}=0.15;y_{2,5}^{1}+y_{4,5}^{1}+0.10-y_{5,2}^{1}-y_{5,4}^{1}-y_{5,8}^{1}\geq 0.20;\\ 0\leq y_{0,2}^{1}+y_{3,2}^{1}+y_{5,2}^{1}+y_{6,2}^{1}+y_{7,2}^{1}+0.20-y_{2,3}^{1}-y_{2,5}^{1}-y_{2,6}^{1}-y_{2,7}^{1}\leq 0.15;\\ 0\leq y_{0,3}^{1}+y_{2,3}^{1}+y_{4,3}^{1}+y_{6,3}^{1}+0.20-y_{3,2}^{1}-y_{3,4}^{1}-y_{3,6}^{1}\leq 0.15;\\ 0\leq y_{0,4}^{1}+y_{3,4}^{1}+y_{5,4}^{1}+y_{6,4}^{1}+y_{7,4}^{1}+0.20-y_{4,3}^{1}-y_{4,5}^{1}-y_{4,6}^{1}-y_{4,7}^{1}\leq 0.15;\\ y_{1,7}^{1}+y_{2,7}^{1}+y_{4,7}^{1}+0.05-y_{7,2}^{1}-y_{7,4}^{1}-y_{7,9}^{1}\geq 0.15;\\ y_{2,6}^{1}+y_{3,6}^{1}+y_{4,6}^{1}+0.05-y_{6,2}^{1}-y_{6,3}^{1}-y_{6,4}^{1}-y_{6,9}^{1}-y_{6,8}^{1}\geq 0.15;\\ y_{2,3}^{1}\times y_{3,2}^{1}=0;y_{2,6}^{1}\times y_{6,2}^{1}=0;y_{3,6}^{1}\times y_{6,3}^{1}=0;y_{3,4}^{1}\times y_{4,3}^{1}=0;y_{4,7}^{1}\times y_{7,4}^{1}=0;y_{4,5}^{1}\times y_{5,4}^{1}=0;\\ y_{4,6}^{1}\times y_{6,4}^{1}=0;y_{3,6}^{1}\times y_{6,3}^{1}=0;y_{1,7}^{1}\times y_{7,1}^{1}=0;y_{2,7}^{1}\times y_{7,2}^{1}=0;y_{2,5}^{1}\times y_{5,2}^{1}=0;\\ 0\leq y_{i,j}^{r(i,j)}\leq h_{i,j}^{r(i,j)};0\leq y_{j,i}^{r(j,i)}\leq h_{j,i}^{r(j,i)},i,j\in \left(0,1,2,3,4,5,6,7,8\right) \end{array}\right.$$

**Figure 9:**
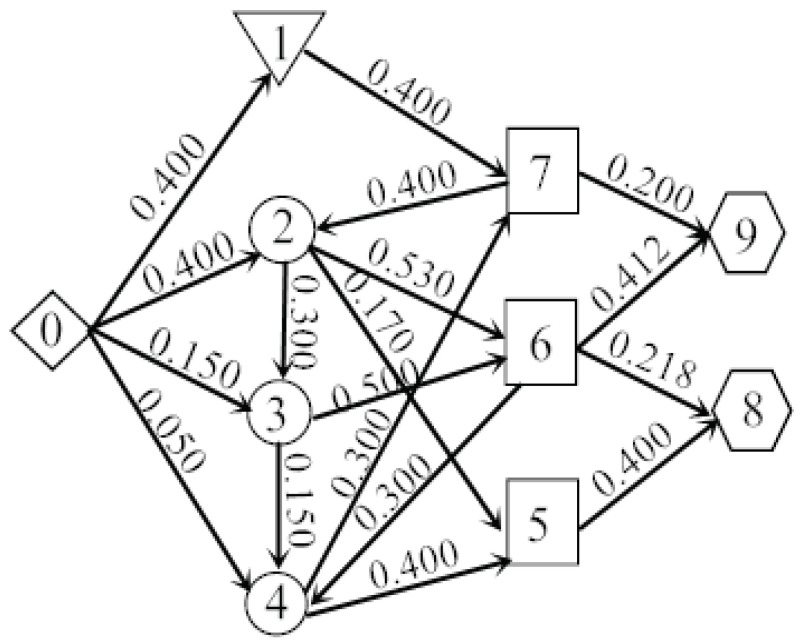
Military supply optimal flow distribution.

The concealment measure of military supply transportation is constructed according to the objective function and the constraint conditions. The circulation flows are removed in the process of calculation to maximize the concealment degree and obtain the optimal solution. The calculated results of $y_{ij}$ in the example can be found in Table 2. The specific flow distribution could be seen from Figure 9.

In Figure 9, there are different shapes represent different types of nodes in the road network. The rhombus and the pentagons are the starting point and endpoints respectively. The inverted triangle, the circles and the squares are used to represent different relationships between inflows and outflows in the transit points. The inverted triangle means the inflows are equal to the outflows, the circles mean the inflows are less than the outflows and the squares mean the inflows are more than the outflows.

Additionally, the rest of military supplies in each node can be calculated, which are $G^{\prime }=\left(g_{1}^{\prime },g_{2}^{\prime },g_{3}^{\prime },g_{4}^{\prime },g_{5}^{\prime },g_{6}^{\prime },g_{7}^{\prime },g_{8}^{\prime },g_{9}^{\prime }\right)=(0.150,0,0,0,0.270,0.150,0.150,0.618,0.612)$.

### 5.2. Result Analyses

According the above calculations and analysis, we can find that the concealment degree and circulation flows in military supply transportation are considered, which are two obvious advantages compared with other similar measures and methods. Thus, the proposed approach is beneficial as it can provide the optimal scheme which maximizes the concealment degree and time efficiency.

Moreover, based on the above calculations and results, we can further derive more detailed conclusions which also demonstrate the effectiveness and superiority of the proposed methods as follows:

(1) When some road sections have the same length and the same maximum traffic flow, the principle “the more lanes the greater effectiveness” is verified by the calculated results, that is, the points 0 to 2 can transport more military supplies than the points 0 to 4 can do due to the number of lanes.

(2) Due to the circulations in the flow distribution, the efficiency of the road network will decrease. So in the step of calculating the optimal flow distribution, we can firstly find the circulations in the process of flow distribution, and then introduce the above equations to remove them step by step. Thus, there is no circulation in the final results in Figure 9 which proves the approach could effectively remove the circulations.

(3) The military supply transportation in the road sections that connect the starting point and endpoint are directional, so there are only outflows from the starting point 0 and only inflows to the endpoints 8 and 9.

Here, it should be noted that the proposed model is utilized to analyze the issue of the military supply transportation and its optimal flow distribution when merely knowing about the road conditions and the node types. The optimal flow distribution model can be a theoretical basis for the research of military supply transportation, it also can provide effective and practical schemes in real situations.

## 6. Conclusions

This study has proposed a modified concealment measure and considered different nodes in the road network. By analyzing the nodes qualitatively and quantitatively, finding the constraints of the optimal flow distribution, investigating the assumptions and the objective function, the optimal flow distribution model of military supply transportation has been constructed. Lastly, an example of military supply transportation in Jiangsu province has proved the feasibility and validity of the modified measure and the proposed optimal flow distribution model. The scientific value of this study is that the concealment of military supply transportation can be measured by introducing entropy theory, which is also a creative way to further provide the optimal schemes that maximize the concealment degree. Besides, different types of nodes and inventories are analyzed in detail to minimize the time gap between the steps of material preparation and material supply. Therefore, it is believed that the proposed model in this study is feasible for real-world military supply transportation. Besides, this model can be also utilized in other transportations fields that require concealment, such as antique or works of art transportations.

However, there are still some aspects need to be improved. For instance, this paper mainly focuses on the inventories of the nodes in military supply transportation, but other elements such as traffic jams, weather conditions or other emergencies are not taken into account. In the future research, it is expected that other external conditions are involved to further improve the flow distribution model in the context of military supply transportation.

## Figures and Tables

**Table 1:** Explanation of the involved parameters.

Decision Variables	Description
*i*	A node in the network, i∈(0,1,⋯,V).
*j*	A node in the network, j∈(0,1,⋯,V+1).
$R\left(i,j\right)$	The number of road sections from *i* to *j*.
$r\left(i,j\right)$	One of the road sections.
$F_{i,j}^{r(i,j)}$	The traffic flows on each road section.
*F*	The traffic flows on all road sections.
$s_{i,j}^{r(i,j)}$	The length of the road $r\left(i,j\right)$ from *i* to *j*.
$y_{i,j}^{r(i,j)}$	The standard traffic flows.
*x*	The standard traffic flows on a single-lane road in the unit distance
*k*	The *Boltzmann Constant* and k≥0.
$q_{i,j}^{r(i,j)}$	The number of lanes of the road from *i* to *j*.
*L*,...,*T*,V−M+1,...,*V*	The nodes in road network.
$g_{j}$	The initial inventory in point *j*.
$t_{1}^{j}$	The amount of supplies delivered to the point *j*.
$t_{2}^{j}$	The amount of supplies delivered out from the point *j*.
*t*	The *t* in $y_{j,t}^{r(j,t)}$ represents a node in the network t∈(1,⋯,V+1).
*h*	The maximum traffic flows.

**Table 2:** The optimal flow distribution results in different nodes.

*i*	0	1	2	3	4	5	6	7	8	9
0		0.400	0.400	0.150	0.050					
1								0.400
2				0.300		0.170	0.530			
3					0.115		0.500	
4						0.400		0.300		
5									0.400
6					0.300				0.218	0.412
7			0.400							0.200
