# Correction: Zhou et al. Optimal Flow Distribution of Military Supply Transportation Based on Network Analysis and Entropy Measurement. *Entropy* 2018, *20*, 446

**DOI:** 10.3390/e26030247

**Published:** 2024-03-11

**Authors:** Wei Zhou, Jin Chen, Bingqing Ding

**Affiliations:** 1 Business School, Yunnan University of Finance and Economics, Kunming 650221, China; zhouwei907@ynufe.edu.cn (W.Z.); monkiis@163.com (J.C.); 2 Business School, East China University of Science and Technology, Shanghai 200237, China

In the original publication [1], there was a mistake in Figure 5 as published. 

First and foremost, the authors sincerely apologize for the confusion this error has caused. Upon review, the authors have confirmed that there was indeed a mistake, as Figure 5 should not be identical to Figure 4. This error occurred due to a file management oversight during the final editing process. 

The correct Figure 5 appears below, depicting that the inflows are greater than the outflows. Thus, there should be one more inflow arrow compared with those in Figure 4. 

The authors state that the scientific conclusions are wholly unaffected. This correction was approved by the Academic Editor. The original publication has also been updated.

## Figures and Tables

**Figure 5:**
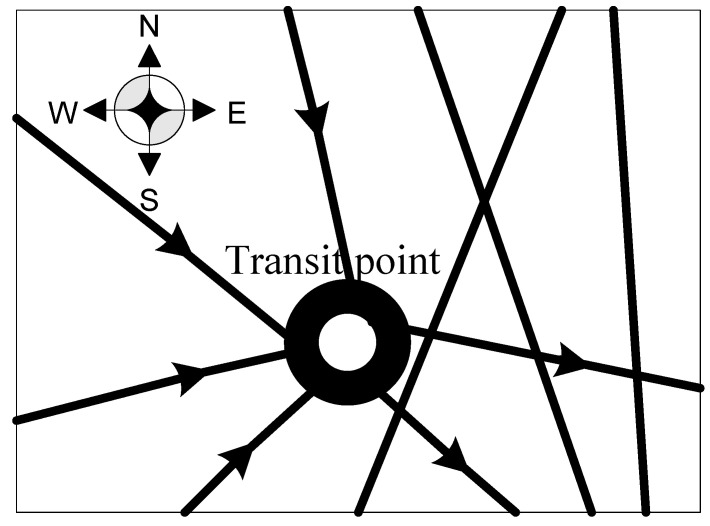
The transit point that inflows are more than the outflows.

## References

[1] Zhou W., Chen J., Ding B. (2018). Optimal Flow Distribution of Military Supply Transportation Based on Network Analysis and Entropy Measurement. Entropy.

